# Spatial distribution characteristics and influencing factors of milk tea stores in Wuhan based on sDNA and OPGD models

**DOI:** 10.1371/journal.pone.0319075

**Published:** 2025-03-11

**Authors:** Wentao Yang, Xinrui Zhan, Dinghui Liu, Huade Zhu

**Affiliations:** 1 College of Urban and Environmental Sciences, Hubei Normal University, Huangshi, Hubei, China; 2 Research Center for the Transformation and Development of Resource-Depleted Cities, Hubei Normal University, Huangshi, Hubei, China; Chang'an University, CHINA

## Abstract

Milk tea stores have rapidly expanded in Wuhan due to residents’ increased consumption demand. Therefore, studying the spatial distribution and influencing factors of stores is important for optimizing their layout and promoting economic development. Using milk-tea store data from Amap City’s Point of Interest function and road network data from Baidu HeatMap, we analyzed the spatial distribution characteristics of stores within the third ring road of Wuhan City using ArcGIS. We then examined the influencing factors by combining spatial design network analysis, optimal parameters-based geographical detection, and location-based service big data. Our results revealed the following: (1) The spatial distribution of stores was concentrated in areas with high closeness and betweenness centrality, forming a multi-core “northwest–southeast” distribution pattern with significant spatial positive correlation. (2) The stores’ spatial pattern was influenced by the road network betweenness and the presence of office buildings, shopping malls, shopping centers, and tourism resources. The road network betweenness had the greatest impact on the stores’ spatial distribution, while the kernel density of betweenness presented a “one major and multiple sub-core” structure consistent with that of the stores’ spatial distribution. The kernel density of closeness and betweenness regulated the formation of the stores’ core area and multiple sub-core areas, respectively, and both factors governed the stores’ spatial distribution, which was characterized by a “widely-scattered and sporadically-clustered” pattern. (3) The stores’ distribution was closely associated with the spatial and dynamic population distribution at different times of the day. By demonstrating the big data for the spatial distribution and driving factors of milk tea stores at the urban regional scale, we fill the research gap on the spatial distribution of milk tea stores at the meso-scale. Our results offer insights into the future urban planning of milk tea stores amid the current milk tea craze.

## Introduction

In recent years, the milk tea industry has seen an unprecedented economic expansion of new tea drinks. The China Chain Store Management Association’s New Tea Drinks Research Report revealed 486,000 new tea drink stores in operation in China in 2022, representing a 28% increase from 2020, with the value of the new tea drink market projected to reach RMB 145 billion by 2023 [1]. Alongside the accelerated growth of the milk tea economy, consumers are engaging in considerable “milk tea traffic” in their daily lives, including the Internet phenomenon of consuming “the first cup of milk tea in autumn,” the proliferation of the “Mixue theme song,” and the occurrence of “queuing for eight hours to buy Sexy Tea” events. Compared to the traditional beverage market, the milk tea market has a distinct brand identity, uses diversified marketing strategies and communication methods, and focuses on personalized needs to reflect both consumer behavior and consumption patterns in the contemporary fast-paced environment [2–3]. Milk tea products, owing to their rich and unique materiality (such as packaging and store decoration), possess an intrinsic value that extends beyond their worth as a beverage. Further, milk tea’s symbolic consumption scene is highly sought after by young people [4–6].

Milk tea, a mixture of milk and tea, originates from Mongolian grassland tea. It spread to other regions during the Yuan dynasty and was further developed abroad, such as in the United Kingdom and the Netherlands, to evolve into the beverage known today as milk tea [7]. The domestic research on milk tea has investigated its development from the historical, economic, tourism, social, and food science perspectives [8]; the evolution of marketing models[9]; industry growth [10–11]; and the role of consumer and consumption symbols [12–13]. However, from the geographical perspective, domestic research on milk tea remains in its infancy, with most studies focusing on store locations, spatial patterns, and consumption spaces. For example, Huang et al. [14] examine the factors contributing to the economic prosperity of milk tea and offer insights from the economic and cultural geographic perspectives. Huang et al. [15] analyze the spatial distribution of Sexy Tea stores in Changsha City using a random forest model and reveal the location and adaptability of such stores. Lin et al. [16] employ spatial autocorrelation and hotspot analysis methods to examine the spatial distribution patterns of different brands’ milk tea stores; they reveal that such stores’ spatial distribution dynamics are shaped by brand positioning, regional cultural episodes, and economic levels. Finally, Zhang et al. [17] analyze the distribution characteristics of milk tea consumption in Xi’an using a threshold regression model and multi-scale geographically weighted regression model, and reveal the production and reproduction mechanisms of new retail consumption spaces.

Traditional consumer space research views space as an urban node, a container for economic and social development, and considers space as a backdrop for human activity[18–19]. In recent years, with the development of information technology, some scholars have begun trying to integrate and superimpose the physical space and information space by space syntax theory to expand the research dimension of consumption space[20–21].

Space syntax theory pertains to the relationship between spatial organization and human society through quantitative descriptions of spatial structures [22]. Many Chinese scholars in the field of space syntax have gradually shifted focus from its initial architectural design [23], urban structure [24], road network morphology [25], and other related areas, to encompass a more diverse range of topics including tourism [26–27], planning and protection of traditional villages [28–29], and commercial sites [30]. The application of spatial syntax is becoming increasingly integrated with geographic information systems (GIS) [31–32]. The spatial design network analysis (sDNA) model has become popular among scholars because of its reliance on the ArcGIS platform and its superior computational capabilities and stability when compared with traditional space syntax software. Moreover, its rigorous search radius algorithm allows for a more comprehensive analysis by simultaneously considering topology, economics, and other aspects, thus enhancing the algorithm’s spatial syntax. It also has the potential to enhance the modelling and analytical capabilities of space syntax in urban and large-scale regions [33]. Nevertheless, there is a paucity of empirical studies on sDNA in China, and even fewer applications in geostatistics [34–35]. Some domestic studies include Gu et al. [36], who employ sDNA and geographically-weighted regression models to undertake a spatiotemporal analysis of residential prices based on the road network pattern in Guangzhou City, resulting in the proposal of a network-based hedonic model. Lin et al. [37] employ the sDNA-modified gravity model to quantify and examine the interaction intensity and evolution characteristics of the urban–rural transition zone in Guangzhou City, and its relationship with socioeconomic development. Finally, Liu et al. [38] construct an origin-destination cost matrix with an sDNA syntactic analysis model to calculate the road network accessibility of Guiyang city center.

In conclusion, scholars have employed sDNA models to examine the spatial configuration and motivation of milk tea stores in China; however, the number of milk tea store brands selected for analysis is limited. Further, there is a paucity of research on the distribution of milk tea stores at the meso-scale, while the impact of human flow on the distribution of milk tea stores has not been considered. Even if you do not drink milk tea, strolling along the streets of Chinese cities, you will find different grades and kinds of milk tea stores filling up the consumption space of the city. The large number of milk tea spaces caters to the general trend of transformation from production space to consumption space in Chinese cities. Therefore, studying the distribution characteristics of milk tea stores, a new type of consumption space, and its influencing factors from a meso-scale is an important reference for the layout and planning of new retail consumption spaces in cities. In the era of the digital economy, the influence of new retail consumption space, which is the fusion and superposition of urban physical space and information space, and the joint role of scene consumption and symbolic consumption, on the development of urban space is expanding, but related research remains scarce. Therefore, on the basis of existing research, this study takes Wuhan as an example, considers the influence of material and information flows such as road network and pedestrian flow on the distribution of milk tea stores, and explores the distribution characteristics of milk tea consumption space in this new spatial production mechanism.

To fill these gaps, we consider the influence of road networks and pedestrian flow on the spatial distribution of milk tea stores in Wuhan City. We then use an sDNA model, optimal parameters-based geographical detection (OPGD), and location-based service (LBS) big data to explore the influencing factors of the stores’ distribution at the meso-scale based on three perspectives: the stores’ spatial distribution characteristics, influencing factors, and thermal population coupling. This paper provides an empirical evidence base for spatial distribution and driving factors of milk tea stores at the regional city level. It also addresses a gap in research on the spatial distribution of milk tea stores from the meso-scale perspective. Furthermore, the paper provides a reference point for the future planning and layout of milk tea stores in cities based on the context of the current phenomenon of the “milk tea frenzy”.

## Method

### Research outline

**Fig 1:**
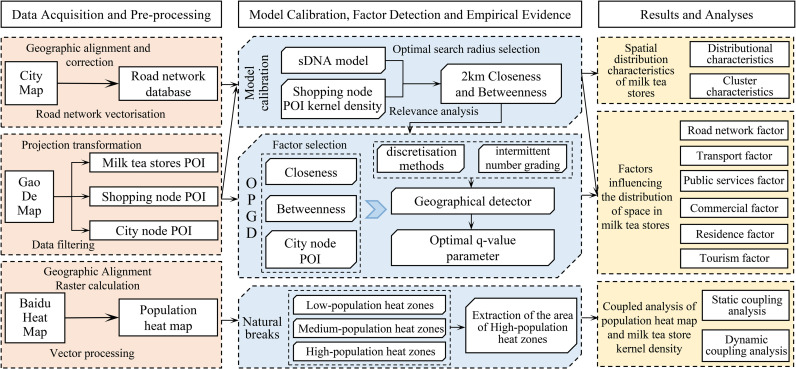
Outline for studying the spatial distribution characteristics and influencing factors of milk tea stores.

First, we established and calibrated an sDNA model within the study area (Wuhan City) to select an optimal search radius. We then analyzed the spatial distribution and clustering characteristics of the milk tea stores using ArcGIS. Second, based on the extant studies [15,17], we identified the geographic detector factors affecting the distribution of milk tea stores and determined the optimal parameters of each factor using various discretization methods and intermittent number grading. We used an OPGD to study the factors influencing the stores’ spatial distribution. Third, we used the natural breakpoint method to categorize the population heatmap into low-, medium-, and high-density zones. We then analyzed the stores’ distribution in conjunction with the population heatmap, both statically and dynamically. Our research approach is illustrated in Fig 1.

### Study area and data sources

To circumvent the boundary effect, our study area comprised the central urban area within the complete closed-loop urban expressway (third ring road) in the main urban area of Wuhan City. This area encompassed the Jiangan, Jianghan, Qiaokou, Hanyang, Wuchang, Qingshan, and Hongshan districts alongside part of the East Lake Hi-Tech Zone, and has a 104.76 km perimeter and an area size of 709.77 km².

**Fig 2:**
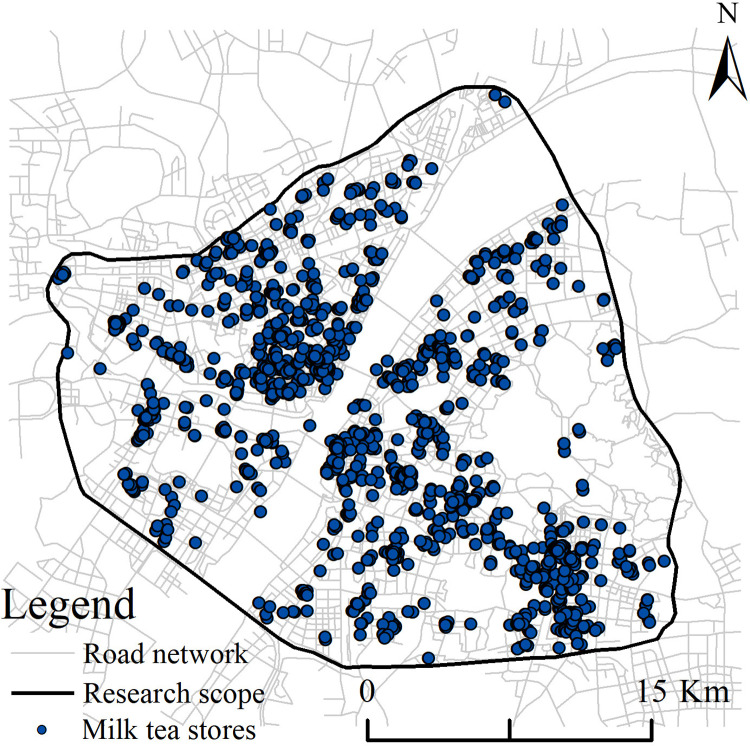
Schematic diagram of the study area.

The road network map of Wuhan used in this study was obtained freely from the Standard Map Service website of the Ministry of Natural Resources of China (http://bzdt.ch.mnr.gov.cn/index.html) In China, maps from this source can be used in academic publications without explicit permission, as clarified in a public statement issued by the Ministry of Natural Resources of China. We obtained the data on milk tea stores and other urban data from the points of interest (POI) data on the 2022 Gaode Map Open Platform (https://lbs.amap.com/). These were then projected and corrected for coordinates prior to being used as our research data. In the POI classification system, the milk tea stores were classified under the umbrella term of “cold drink shops.” Overall, we identified 2,751 cold drink shops in Wuhan City, which we filtered individually to obtain 1,612 milk tea stores within the third ring road (Fig 2).

### Research methodology

#### Kernel density estimation (KDE).

We employed the KDE to reveal the spatial distribution of the milk tea stores and road network syntax parameters in the study area. This method enables the calculation of the data clustering and dispersion degrees across an entire area based on the sample data. Higher density indicates a greater degree of spatial clustering among the samples. Points or lines within a specified search radius are assigned various weights. An assigned weight is inversely proportional to the distance from the center of the search radius. This results in the generation of a continuous density surface, which we calculated using the following formula [39]:


$$\int_{n}(x)=\frac{1}{nh}\sum_{i=1}^{n}k(\frac{x-x_{i}}{h})$$ (1)

where the kernel function (*k*) is a mathematical operation that determines the density of a given point (*x*) relative to its surrounding area; bandwidth (*h*), also known as the search radius, defines the extent of the surrounding area; and the distance between the estimated point *x* and reference point *xi* is represented by (*x*-*x*_*i*_). The KDE of point *x* is the result of the formula. In practice, the choice of bandwidth (*h*) can significantly impact the outcome. A smaller *h* value may result in an overly abrupt transition between the mapped density and surrounding area, whereas a larger value may obscure some of the finer calculation details [40]. The results of several comparison tests indicated that 1,200 m provided a more accurate reflection of the density center of the milk tea stores on the third ring road.

#### Standard deviational ellipse (SDE).

We used the SDE to quantify the directionality of the sample’s distribution. This method comprises three principal elements: the angle of rotation, denoted by θ; the standard deviation along the long axis; and the standard deviation along the short axis. The long axis of the ellipse represents the direction with the highest spatial distribution, whereas the short axis represents the direction with the lowest spatial distribution [41–42]. We used the SDE to reflect the spatial distribution directionality of the space syntax parameters of the milk tea stores and road network.

#### Spatial autocorrelation.

We used global autocorrelation to ascertain the aggregation of the milk tea stores across the entire spatial domain [43]. We used local autocorrelation to examine the regions wherein the milk tea stores were concentrated, as follows [44]:


$$I_{i}=z_{i}\sum_{j\neq i}^{n}w_{ij}z_{j}$$ (2)

where the spatial values *z*_*i*_ and *z*_*j*_ are normalized, *w*_*ij*_ is the spatial weight matrix; and the *I*_*i*_ value is positive when the spatial unit is consistent with the attributes of neighboring spatial units (high-high: H-H, low-low: L-L) and negative when it is not (high-low: H-L, low-high: L-H).

#### LBS big data.

LBS big data is used to describe the utilization of mobile smart terminals to ascertain a user’s geographic location, which is then relayed to a corresponding service platform. This enables the provision of services to the user via GIS [45]. The Baidu Heat Map is a visual representation that illustrates the spatial and temporal population distribution by obtaining users’ geographic locations on the LBS Baidu platform and employing a color-coding system. The proximity to a warm color indicates higher population concentration and density in a region. Conversely, the proximity to a cold color signifies a more dispersed population distribution and a lower population density. We then employed a population heatmap to investigate the consistency between the spatial distribution of the concentrated population areas and milk tea store areas. We used Baidu HeatMap data from March 11 (Saturday) and 13 (Monday) of 2023 as the basis for the analysis. Our sample period was from 06:00–00:00, with intervals of 2 h, resulting in 20 heatmaps for the 2 week and weekend days. We then geographically aligned, raster computed, and vector processed the heatmaps using ArcGIS and classified seven categories using the natural discontinuity method. We designated categories 1–3 as low population heat zones, categories 4–5 as medium population heat zones, and categories 6–7 as high population heat zones. Additionally, we computed the coupling areas of the high population heat zones and high heat zones with the high nuclear density of the milk tea stores.

#### Extended space syntax.

The evolution of space syntax theory has resulted in the emergence of numerous models. Among these, axis and line segment models have gained attention in the human geography domain [46]. The line segment model is more realistic in its simulation of human travel patterns as it considers factors such as metric distance and street deflection angle. It also enables a comparison of syntactic parameters under different radii [47]. We employed an sDNA model to analyze the correlation between closeness, betweenness, and the urban functional area KDE of the road network in the study area under different radii. We used the road network radii with the highest degree of fit to analyze the factors influencing the spatial distribution of the milk tea stores [32,48]. The closeness metric indicates the difficulty of a given road in accessing other roads within a specified search radius, so as to reflect the accessibility of the road in question, which was modelled as follows [49–50]:


$$NQPDA\left(x\right)=\underset{y\in rx}{\sum }\frac{p\left(y\right)}{d\left(x,y\right)}$$ (3)

where *NQPDA(x)* represents closeness, *p(y*) denotes the weight of point *y* within the search radius *r*, and *d(x, y*) signifies the shortest angular topological distance from *x* to *y*. In continuous space, *p(y)* is constrained to the range [0,1], whereas in discrete space, it can assume either the value of 0 or 1.

We used the betweenness metric to measure the frequency in which paths were chosen in the road network, so as to reflect the road network’s choice for the road in question, which was modelled as follows [33,36]:


ODy,z,x=0,Othersituations1/3,x≡y≡z1/2,x≡y≡z1/2,x≡y≡z1,Theshortestroutefrompointytopointzpassesthroughpointx. (4)

$$TPBt\left(x\right)=\sum_{y\in n}\sum_{z\in rn}OD\left(y,z,x\right)\frac{P\left(z\right)}{links\left(y\right)}$$ (5)

where *tpbt(x)* represents betweenness, and *OD(y, z, x)* denotes the shortest angular topological distance between *y* and *x* through point *z*. The variable link (*y*) is defined as the total number of nodes within a search radius centered at *y*.

#### OPGD.

Geographical detectors represent a novel statistical analysis method for identifying and elucidating geospatial dissimilarities and their underlying causal factors [51]. The OPGD represents an advancement over the geographical detector because it can circumvent the shortcomings associated with poor discretization outcomes and the inherent subjectivity of continuous variables. We processed the data using multiple discretization methods (equal, natural, interquartile, geometric, and standard deviation intervals) and graded the number of intervals from 3–10. We used a combination of discretization methods and intervals with the highest factor detection values. We then applied the OPGD to detect the intensity of different influencing factors on the distribution of the milk tea stores, which was modelled as follows [52]:


$$q=\text{1}-\frac{\sum_{h=\text{1}}^{L}N_{h}{\sigma_{h}}^{2}}{N\sigma^{2}}$$ (6)

where *q* represents the detection value of the factor affecting the distribution of milk tea stores, *N* denotes the number of milk tea stores within the study area, *L* signifies the partition of the influencing factors, and *σ2* denotes the distribution density variance of the milk tea stores. The value range of *q* is [0,1], and a larger value indicates that the influencing factor has greater explanatory power regarding the density of milk tea stores. We established a 1 km buffer zone around each milk tea store, with the number of other POI types falling within the buffer zone. We used the average number of POIs in the buffer zone of each milk tea store on the road grid as the influencing factor. Based on the extant studies [53–55], we selected road network syntax parameters, transportation locations, public services, and tourism resources as the influencing factors and analyzed their impacts on the milk tea stores’ distribution. Ultimately, we selected six categories of influencing factors (totaling 15 influencing factors) as the research indicators (Table 1).

**Table 1:** Influencing factors of the spatial distribution of milk tea stores.

Influences	Influencing factors
Road network factor	Closeness, betweenness
Transport factor	The average number of metro stations, train stations, passenger terminals, and bus stops within the designated buffer zone.
Public services factor	The mean number of colleges and universities, elementary and secondary schools, hospitals, and parks within the buffer zone.
Commercial factor	The mean number of shopping malls, shopping centers, and office buildings within the buffer zone.
Residential factor	The mean number of living areas within the buffer zone.
Tourism factor	The mean number of tourism resources within the buffer zone.

## Empirical analysis

### Calibration and selection of the sDNA model’s search radii

We considered the boundary effect inherent to the space syntax model as well as the potential for discrepancies in the search radii to influence the spatial syntax calculation outcomes. Therefore, following the extant studies [32,48], we calibrated the sDNA model in terms of the range and search radii. To delineate the modelling scope, we adhered to the principle of moderation by extending the study boundary outward by a buffer zone of 1,600 m and incorporating the roads within this zone into the model.

To select the search radii, we considered the following distances: 0.4 km, 0.8 km, 1.2 km, 2 km, 5 km, 8 km, and 10 km, based on residents’ typical travel modes and times (Table 2). Typically, search radii selection and calibration are achieved using the traditional traffic flow calibration method. This involves correlating the calculated space syntax roadway parameters with the actual statistical traffic flow of the roadway with the optimal radius selected. However, the advent of big data has opened new avenues for exploring the calibration of space syntax models, and the prior research has demonstrated a high degree of consistency between commercial nodes and space syntax closeness in POI big data [48]. Consequently, we examined the relationship between the POI shopping node KDE and space syntax parameters after converting them to surface data, so as to identify the optimal radius of the road network that influences the distribution of the milk tea stores.

**Table 2:** Relationship between pedestrian travel mode, time, and distance.

	0.4 km	0.8 km	1.2 km	2 km	5 km	8 km	10 km
Walk	5 min	10 min	15 min	25 min	62.5 min	100 min	125 min
Drive	–	–	–	4 min	10 min	16 min	20 min

**Fig 3:**
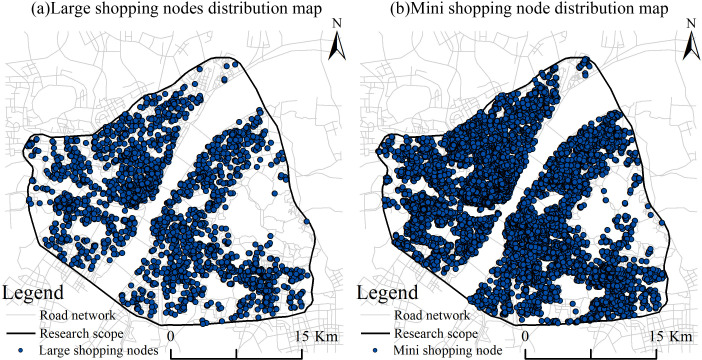
Distribution of POI shopping nodes. (a) Large shopping node distribution map. (b) Small shopping node distribution map.

We obtained 119,280 data points for large shopping nodes (i.e., shopping centers, supermarkets, and malls) and small shopping nodes (including convenience supermarkets, bazaars, and stores) within the study area (Fig 3). We then calculated the KDE of the two node types and converted the results into faceted data. We further computed the closeness and betweenness of the sDNA model for the different search radii. Finally, we obtained the correlation coefficients by correlating the two using SPSS software (Table 3).

**Table 3:** Relationship between space syntax parameters and POI shopping node KDE under different search radii.

		0.4 km	0.8 km	1.2 km	2 km	5 km	8 km	10 km
Closeness *NQPDA* (*x*)	Large shopping nodes	0.478	0.565	0.619	0.668	0.572	0.466	0.467
	Small shopping nodes	0.603	0.693	0.747	0.800	0.681	0.562	0.569
Betweenness *TPBt* (*x*)	Large shopping nodes	0.371	0.378	0.385	0.378	0.255	0.169	0.137
	Small shopping nodes	0.460	0.462	0.461	0.439	0.270	0.156	0.121

The results demonstrate that (1) compared with the large shopping nodes, there is a higher correlation between the small shopping nodes and space syntax parameters of the road network, suggesting that small shopping nodes (e.g., convenience stores) are more susceptible to the influence of the road network. (2) Using the same radius scale, the correlation of the shopping node closeness is typically greater than that of the choice, suggesting that shopping nodes are more susceptible to the influence of road network accessibility than penetration. This finding implies that shopping nodes facilitate greater accessibility in urban areas. (3) A comparison of the space syntax parameters of the road network across different radii reveals an inverted “U” shape with the increase in search radii. The closeness of the two node types reaches its maximum value at 2 km, whereas the betweenness is highest at 1.2 km. This finding suggests that small shopping nodes offer a greater range of services at the meso- and micro-scales of Wuhan City. Considering these findings, we identified the space syntax parameter at the 2 km search radius as a factor influencing the distribution of milk tea stores along the road network.

### Spatial distribution characteristics of milk tea stores

#### Distribution and morphological characteristics.

**Fig 4:**
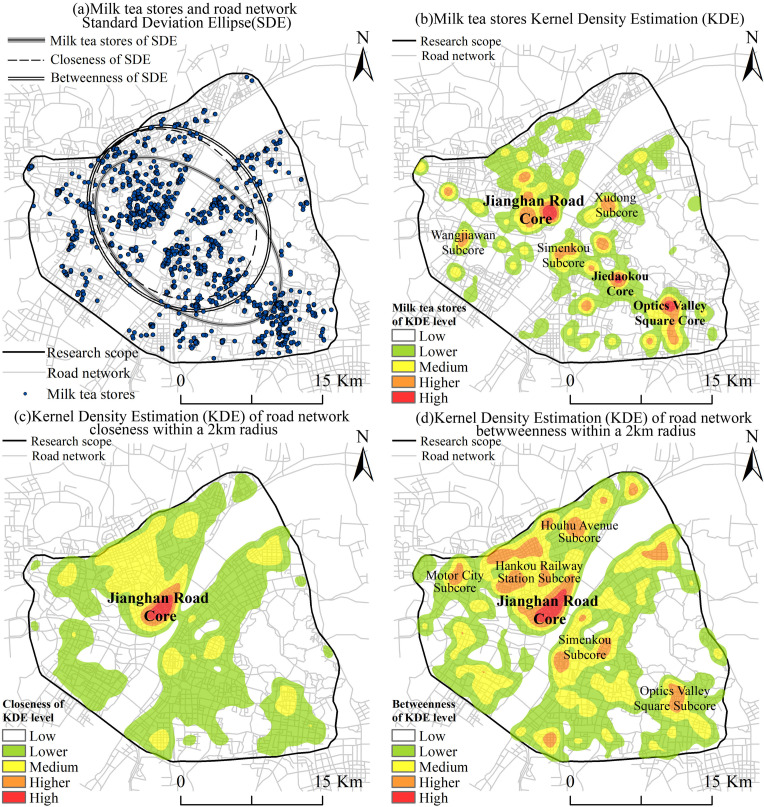
SDE and KDE distributions of the milk tea stores and road network. (a) SDE of milk tea stores and road network distribution. (b) KDE of milk tea stores. (c) KDE of road network closeness within a 2 km radius. (d) KDE of road network betweenness within a 2 km radius.

Fig 4(a) illustrates the SDE of proximity and closeness within a 2 km radius of the milk tea stores. The results demonstrate that (1) the spatial distribution of milk tea stores and the proximity and closeness of the road network exhibit a similar orientation that is aligned with the “northwest–southeast” direction. This suggests that the stores’ distribution is somewhat influenced by road network accessibility and choice. The dataset does not allow for a choice to be made. (2) The SDE of the milk tea stores is longer than that of the syntactic parameters of the road network in terms of the shape of the long axis, while the short axis is more convergent. Overall, it is more concentrated in the “northwest–southeast” direction, forming a multi-core distribution pattern along the ellipse’s long axis. The milk tea stores’ distribution is influenced by the road network and exhibits a greater aggregation degree in the distribution direction. (3) The SDE of the road network is inclined in the northwest direction in the study area, with the center point located in Jianghan District. Conversely, the SDE of the milk tea stores is more centered in the study area, with the center point situated in Wuchang District. At the 2 km local scale, the road network accessibility and penetration functions exhibit greater concentrations in traditional urban areas, such as Hanyang and Jianghan.

A comparison of Fig 4(b)–(d) reveals that the KDE of the road network closeness forms a single reachable core under the 2 km radius, whereas that of the road network betweenness has a penetrating core comprising one main road and numerous branches. The penetration function for the region as a whole is larger than that of the reachable function. From the distribution perspective, both the reachability and passability factors have significant cores within the 2 km local radius near the Jianghan Road area. The passability continuity of the roads in the region is superior to that of the reachability factor. The SDE analysis corroborates these findings, indicating that the roads’ closeness is superior to that of their betweenness in terms of the number of cores and SDE size. This suggests that the roads’ reachability exceeds that of their passability within a local radius, and that the cohesion characteristics exert a shaping influence on the clustering distribution of the milk tea stores.

#### Spatial agglomeration characteristics.

**Fig 5:**
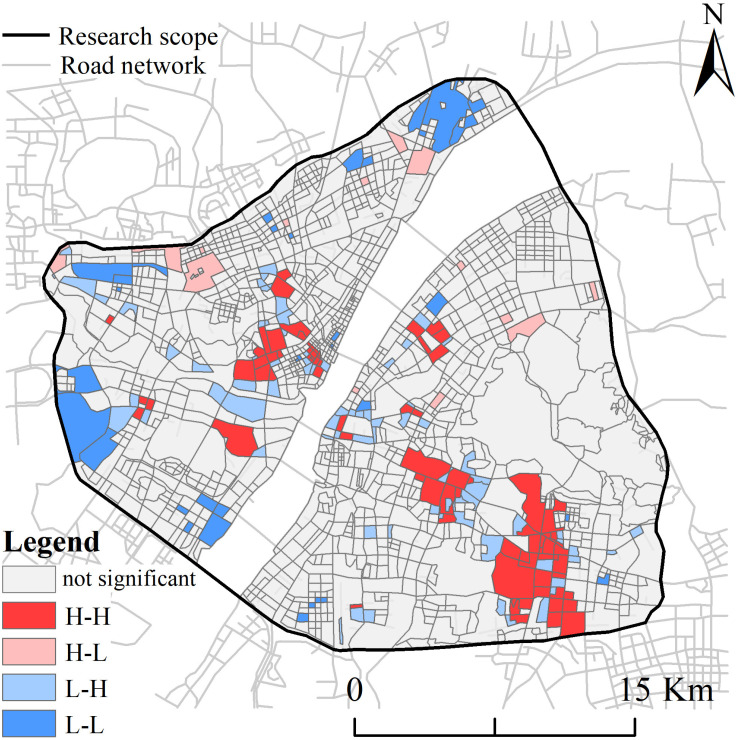
Local spatial association of the milk tea stores.

We used the global and local autocorrelation tools in the GeoDa software to quantify the extent of the milk tea stores’ spatial distribution agglomeration. The global Moran’s index was 0.148, indicating a significant positive spatial correlation and a tendency for the stores to be spatially clustered. We used the local autocorrelation tool to assess the local heterogeneous characteristics of the stores in each road grid. The results (Fig 5) demonstrate that (1) the H-H agglomeration zone is characterized by a concentration of milk tea stores in both the area and surrounding region. Their distribution exhibits a KDE value with an approximate uniform distribution. This phenomenon is particularly evident near Jianghan Road, jiedaokou, and Guanggu Plaza. The clustered distribution of areas containing many milk tea stores indicates the existence of robust economic activity and considerable pedestrian traffic in such areas. This indicates a correlation between the milk tea stores that may manifest as mutual competition or cooperation, which may, in turn, contribute to the clustering of stores in these areas. This also suggests that the region has a higher level of commercial development and market demand, which can attract the relocation of more milk tea stores. As the number of milk tea brands increases and market competition intensifies, the opening of new shops necessitates the consideration of factors such as transport accessibility and traffic flow within the business district. Consequently, areas with the highest concentrations of stores are often economically active and have convenient transport links. (2) The H-L aggregation zone is characterized by a high concentration of milk tea stores within the area and a relatively low concentration in the surrounding region. The number of shops in this zone is low and they are dispersed. (3) The L-H aggregation zone is characterized by a low concentration of milk tea stores in the area but a high concentration in the surrounding region, particularly in the vicinity of the H-H zone. (4) The L-L aggregation zone is characterized by a low number of milk tea stores in both the area and surrounding region. The distribution is mainly concentrated near the third ring road, where the population density and economic activity are relatively low.

### Analysis of factors influencing the spatial distribution of the milk tea stores

**Fig 6:**
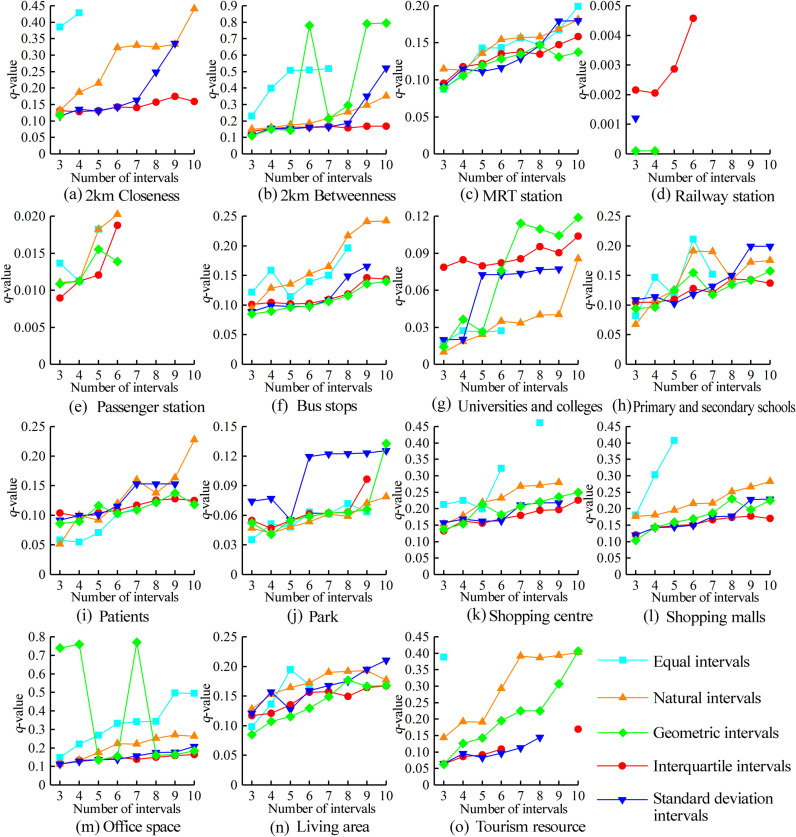
Changes in the *q*-values of different influencing factors. (a) 2 km closeness. (b) 2 km betweenness. (c) Metro stations. (d) Train stations. (e) Passenger terminals. (f) Bus stops. (g) Colleges and universities. (h) Primary and secondary schools. (i) Hospitals. (j) Parks. (k) Shopping centers. (l) Shopping malls. (m) Office buildings. (n) Living areas. (o) Tourism resources.

We used an OPGD to calculate the most appropriate discretization methods and number of breaks for the 15 influencing factors within the 6 categories. This was done to obtain the optimal discretization method and number of breaks, and to determine the optimal *q*-value of each influencing factor (Fig 6).

As illustrated in Fig 6, the impacts of the influencing factors are significantly determined by the choice of discretization method and number of interruptions, as follows. (1) The overall *q*-values of the 15 influencing factors demonstrate an upward trajectory with the increase in number of interruptions. (2) Ten intervals is the optimal number for closeness and betweenness under the road network factor. However, the *q*-value of betweenness exhibits greater variability for the different discretization methods and numbers of intervals. The maximum *q*-value of betweenness is the geometric discontinuity of 10, whereas the minimum *q*-value is the geometric discontinuity of 1, with a difference of 0.685. (3) The *q*-values of the metro stations and bus stops under the transportation factor generally increase with the number of discontinuities, although the iso-intermittency of the bus stops decreases at 5 discontinuities. The *q*-values of the train stations and passenger terminals are notably low and are concentrated between 3 and 6 interruptions. (4) The *q*-values of the four influencing factors under the public services factor demonstrate fluctuating increases with the increase in the number of interruptions. Among these, the geometric interruption of the *q*-value of colleges and universities exhibits a notable surge between 5 and 7. (5) The discrete methods for shopping malls and shopping centers under the commercial factor exhibit similarities in their folding patterns, with their *q*-values under equal intervals displaying elevated levels. The other four interval methods demonstrate a gradual increase with the increase in the number of discontinuities. The geometric discontinuity *q*-value of the office buildings is significantly larger than that of the other discrete methods, exhibiting a pronounced fluctuation with the increase in the number of discontinuities. This is reminiscent of the geometric discontinuity characteristics observed under the road network factor’s betweenness. Conversely, the interquartile and standard deviation discontinuities are relatively less influenced by the number of discontinuities. (6) Compared to the tourism factor, the discrepancies in the *q*-values across the discrete methods under the residential factor are less pronounced but more continuous. The iso-intermittent *q*-value for the tourism factor is observed only at intermittent number 3, whereas the interquartile intermittent number exhibits a break between intermittent numbers 7–9.

Next, we used the 15 influencing factors in the OPGD to ascertain the distribution density of the milk tea stores. We separately calculated the *q*-value of each influencing factor, with a higher value indicating a greater explanatory power on the distribution of milk tea stores. The *p*-value denoted the reliability degree of the *q*-values (Table 4).

**Table 4:** OPGD for the spatial distribution of milk tea stores in the third ring road of Wuhan City.

Influences	Influencing factors	Milk tea stores		OPGD statistics	
***q***	***p***	**Discrete method**	**No. of interruptions**
Road network factor	2 km closeness	0.331	0.00	Natural interval	10
	2 km betweenness	0.796	0.00	Geometric interval	10
Transport factor	Metro stations	0.199	0.00	Isthmus	10
	Train stations	0.005	0.83	Interquartile range	6
	Passenger terminals	0.020	0.35	Natural interval	6
	Bus stops	0.140	0.00	Natural interval	10
Public services factor	Colleges and universities	0.119	0.00	Geometric interval	10
	Primary and secondary schools	0.211	0.00	Isthmus	6
	Hospitals	0.275	0.00	Natural interval	10
	Parks	0.133	0.00	Geometric interval	10
Commercial factor	Shopping centers	0.462	0.00	Isthmus	8
	Shopping malls	0.408	0.00	Isthmus	5
	Office buildings	0.770	0.00	Geometric interval	7
Residential factor	Living areas	0.211	0.00	Standard deviation band	10
Tourism factor	Tourism resources	0.407	0.00	Geometric interval	10

As shown in Table 4, the *q*-values of the factors influencing the spatial distribution of the milk tea stores are ordered as follows: road network betweenness, office buildings, shopping malls, shopping centers, tourism resources, road network closeness, patients, residential areas, primary and secondary schools, metro stations, bus stops, parks, colleges and universities, passenger terminals, and train stations. The road network betweenness and presence of office buildings have the largest *q*-values, indicating that these factors have the greatest influence on the distribution of the milk tea stores. Additionally, the tourism factor demonstrates considerable explanatory power for the distribution of the milk tea stores.

Meanwhile, the explanatory power values of the transport, residential, and public services factors are relatively weak for the distribution of milk tea stores, as follows.

(1) The stores’ distribution in the third ring road of Wuhan City is significantly correlated with the road network’s choice, exhibiting a distinctive “large dispersion and small agglomeration” pattern. Further, the road network betweenness has more pronounced explanatory power for the stores’ distribution than that of the road network closeness. The closeness concept is used to indicate a given road network’s accessibility, whereas betweenness is used to indicate a road network’s choice within the same network. Both the road network’s accessibility and choice attract many consumers to milk tea stores, while the road network’s accessibility core and choice core serve as crucial external drivers of the aggregation and distribution of the milk tea stores. In conjunction with Fig 3, our results reveal that the reachable core exhibits a singular core structure as well as pronounced spatial cohesion characteristics, thereby contributing to the formation of the principal core of the milk tea store density. The extensibility of choice is distinguished by a core structure comprising a primary main core and multiple penetrating cores, which shape the stores’ distribution pattern with multiple sub-cores. The *q*-value of the road network betweenness is 0.796, which is considerably higher than that of the road network closeness. This suggests that milk tea stores offer a wider range of choice services in the city. Compared with a single reachable core, the spatial structure of multiple sub-cores within the walk-through core is more consistent with that of the stores’ distribution structure. This reflects the continuity of the distribution and consumption of milk tea and contributes to the formation of the stores’ distribution pattern, which is characterized by a “large dispersion and small agglomeration” pattern.
(2) The commercial factor significantly influences the stores’ distribution. The milk tea industry’s development is driven by physical and virtual traffic, and brand and event marketing strategies enhance consumers’ recognition of brand value. The mean *q*-value of the commercial factor is 0.547, whereas that of the tourism factor is 0.407. This suggests that the distribution of the milk tea stores is highly consistent with that of both the commercial and tourism factors. The mean *q*-value of the office buildings is 0.77, which is the highest value under the commercial factor. Office buildings are typically situated in commercial centers with high concentrations of employees and consumers, and most of these employees have sufficient economic capacity to consume milk tea. Meanwhile, in the vicinity of office buildings, consumption patterns tend to gravitate toward convenient snacks and beverages. As major consumer destinations in urban areas, shopping malls, shopping centers, and tourist attractions entice many consumers who contribute to the growth of the milk tea economy through their purchasing power. The presence of commercial centers can facilitate the creation of favorable brand images for milk tea stores. The large amount of consumers and foot traffic that frequently visit such centers continually refreshes the stores’ market exposure, which attracts more consumers to visit and consume milk tea and increases overall consumption. The aggregation of stores in commercial centers leads to a clustering effect, which drives the development of milk tea stores in the surrounding region. Further, the market competition among different milk tea brands accelerates the growth of “milk tea events.” The emergence of new brands in the market is driven by multiple factors, including the creation of novel consumption spaces, such as the Tea Face brand event known as “queuing up to buy tea”; the utilization of catchy theme songs to generate consumer interest, as demonstrated by the Honey Snow Ice City brand; and the co-branding of milk tea brands and popular movies, as exemplified by the Nai Xue Tea brand collaboration known as “The Journey of Suzumi Buds.” These strategies have contributed to the rise of these brands as key market players. By leveraging network traffic, commercial collaborations, and the creation of “event enclaves,” among other strategies, such brands attempt to establish consumption scenes and symbols for milk tea that attract consumer recognition and value [14].
(3) Regarding the transportation factor, the metro stations and bus stops exert discernible influences on the stores’ distribution. Conversely, the train stations and passenger terminals have comparatively weak influences, with non-significant *p*-values. Both metro stations and bus stops facilitate the convergence of commuters from diverse demographic backgrounds; however, metro stations exhibit superior efficiency in facilitating transfers and accommodating a larger volume of commuters, and exhibit two distinct commuter peaks throughout the day. This aligns with the milk tea stores’ rapid and convenient service profiles. Most metro stations traverse the city’s primary thoroughfares, with many concentrated in the surrounding commercial districts. Some metro station entrances and exits are situated in dense business areas, fostering vibrant commercial scenes that attract milk tea stores to establish themselves in such locations. However, metro stations exert a more pronounced influence on the stores’ distribution compared to bus stops. While there are milk tea stores located near train stations and passenger terminals that exert a certain influence on the stores’ distribution, these two factors have relatively small values compared to other influencing factors. Consequently, their influence on the stores’ distribution within the third ring road is non-significant. Further, the mean *q*-value for the public services factor is 0.187, whereas that for the residential factor is 0.211. Students represent a significant milk tea consumer group, and the distribution of colleges and universities significantly impacts that of milk tea stores. However, the student population’s limited consumption capacity results in a relatively low density of stores located near colleges and universities. Primary- and secondary-school students also have a certain consumption demand for milk tea, as the *q*-value of primary and secondary schools exceeds that of colleges and universities. This is because the distribution of primary and secondary schools exceeds that of colleges and universities. Meanwhile, hospitals, which are mostly situated in locations with high footfall in urban centers, with convenient transport links and high population density, also exhibit certain similarities regarding the distribution of milk tea stores. Finally, residential areas and parks, which are primarily utilized by residents, exhibit a comparatively limited demand for milk tea. These locations typically have a constrained scale of commercial activities that can be supported, thereby diminishing the impact on the milk tea stores’ distribution.

### Coupled analysis of the population heatmap and milk tea store KDE

**Fig 7:**
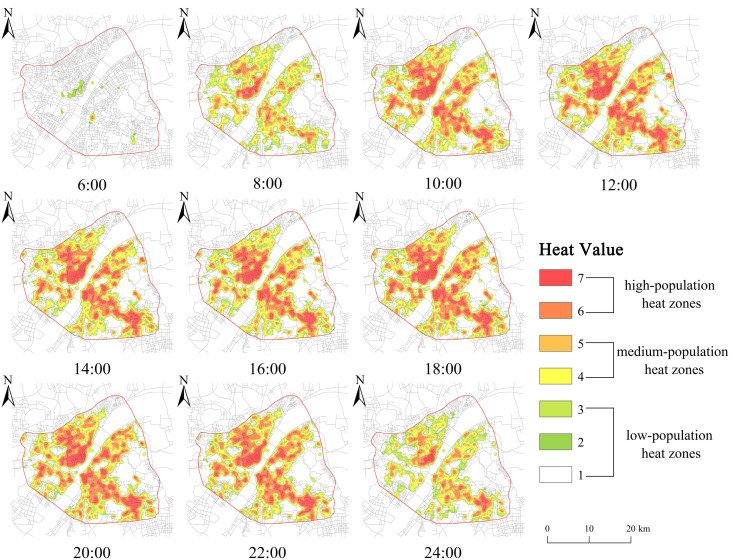
Weekend population distribution heatmap.

We performed a coupled analysis of the population heatmap and milk tea store KDE to examine the correlation between the distribution of the population and milk tea stores. The static coupling analysis results (Fig 7) of the 10 heatmaps and milk tea store KDE on weekends reveal that the high population heat zones are concentrated in Jianghan Road, Hankou Railway Station, Xudong, Simen, Hongshan Square, jiedaokou, and Guanggu Plaza, and exhibit a continuous distribution pattern. The core density of the milk tea store KDE is concentrated in Jianghan Road, Wangjiawan Dayang Department Store, Xudong, Simen, jiedaokou, and Guanggu Square, and exhibits a high coupling degree with the population distribution.

**Fig 8:**
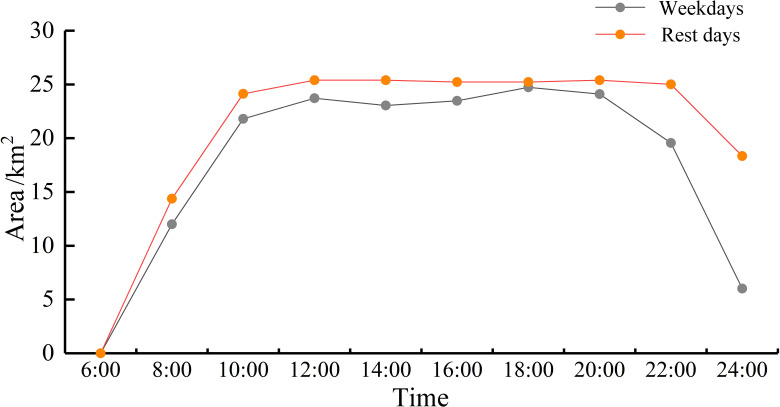
Core density areas of milk tea stores showing the population changes during different times of day.

To provide a more accurate description of the dynamic coupling between the thermal population zone changes and milk tea stores’ core density, we calculated the stores’ core density area using ArcGIS and superimposed the results on the areas of high population heat zones at different times. The results (Fig 8) reveal that the stores’ total core density area is 26.05 km², yielding a map of the changes in the thermal population zones in terms of the stores’ core density at different times. Fig 8 further illustrates the dynamic coupling of weekdays and weekends, which exhibits a “rising–smoothing–declining” trend. The rising interval (between 06:00 and 10:00) is characterized by the gradual accumulation of the population in the stores’ core density area. The subsequent smoothing interval (between 10:00 and 20:00) is characterized by the relatively stable accumulation of the population. The declining interval (between 20:00 and 00:00) is marked by a reduction in population hotspots. The population heat dynamic coupling area on weekdays is slightly lower than that on weekends across all time points, indicating that the population concentration on weekdays is lower than that on weekends. Further, the population heat dynamic coupling area on weekdays declines at 20:00, which is 2 h earlier than the corresponding interval on weekends. The dynamically-coupled areas of the population heat zones during the smoothing interval are all greater than 21 km², exceeding 80% of the total area. This indicates that the core density of the milk tea store KDE is highly coupled with the population heat zones during the smoothing interval.

## Discussion and conclusion

We combined sDNA and OPGD models using ArcGIS software to study the spatial distribution and influencing factors of milk tea stores situated in the third ring road of Wuhan City. We found that (1) the distribution of milk tea stores was concentrated in areas with high road network accessibility and choice, exhibiting a multi-core aggregation pattern. This distribution was more aggregated in the “northwest–southeast” direction. The proximity kernel density under the 2km radius forms a single reachable core, which contributes to the formation of the primary core of the milk tea store kernel density. The intermediate degree exhibits a primary and multiple penetrating core structure that shapes the distribution pattern of multiple sub-cores of the milk tea store. The stores’ distribution exhibited a positive spatial correlation and tendency toward agglomeration. The H-H agglomeration zone was situated close to areas that offered convenient transportation links and developed business districts, such as Jianghan Road, jiedaokou, and Guanggu Plaza. Conversely, the L-L agglomeration zone was predominantly concentrated along the third ring road, which was characterized by a lower population density and relatively limited economic activity.

(2) The influencing factors’ impacts on the stores’ spatial distribution pattern were primarily shaped by road network betweenness and the presence of office buildings, shopping malls, shopping centers, and tourism resources. Betweenness had the most significant influence on the stores’ distribution. The multiple sub-core structure was more consistent with the stores’ distribution structure, which facilitated the formation of the “large dispersion and small agglomeration” pattern. Commercial areas containing office buildings, shopping malls, and shopping centers had high concentrations of employees and consumers, providing excellent opportunities for milk tea stores to create positive brand images and enhance their market exposure to attract more consumers. Further, intensified market competition among milk tea brands enhanced the establishment of “milk tea events.” To create distinctive milk tea consumption scenes and symbols and gain consumer recognition, milk tea stores engaged in marketing activities that utilized network traffic and businesses’ cooperation to create “event enclaves.” The distribution of milk tea stores was not significantly influenced by the transport, public services, or residential factors.

(3) The stores’ distribution was inextricably linked to population distribution. The coupling of the population heatmap and the stores’ core density areas revealed that the latter was concentrated in Jianghan Road, Wangjiawan Ocean Department Store, Xudong, Simen, jiedaokou, and Guanggu Plaza, which was consistent with the high population heat zones. The population hotspot areas exhibited fluctuations throughout the day, displaying a “rising–smoothing–declining” pattern. Further, the population hotspot areas encompassed over 80% of the stores’ core density areas during the smoothing interval, indicating a notable alignment between the population and milk tea stores’ distribution, with a high degree of consistency.

Overall, our results enhance the understanding of the stores’ spatial distribution patterns and influencing factors at the urban scale, and complement the geographic research on the milk tea economy. However, our study had the following limitation. Most milk tea stores use takeaway services. Due to the convenience of milk tea itself and the specificities of milk tea consumer groups, milk tea takeaways increase the spatial service radii of milk tea stores, meaning that their spatial distribution is affected to a certain extent. In the future, our study can be further extended to examine the spatial distribution of milk tea stores under the joint influence of online and offline sales, so as to optimize the stores’ spatial layout.

In today’s consumer society, compared with traditional beverages, milk tea has long exceeded its efficacy attributes as a beverage, and has become a commodity with social, cultural and contemporary attributes that transcend the meaning of food and the realm of daily life. In other words, modern people drink milk tea is not only a material consumption, but also a cultural consumption. Today’s milk tea economy intersects the economic, social and cultural meanings of material consumption, symbolic consumption, social culture, capital operation and market competition. For example, some young people queue up for eight hours just to buy a cup of milk tea called “Cha Yan Yue Shi”. When fall comes, young people will buy a cup of milk tea for their lovers to express their care and love, and milk tea socialization has gradually become fashionable. Even if you don’t drink milk tea, if you walk along the streets of Chinese cities, you will find that different grades and kinds of milk tea stores fill up the consumption space of the city. Consumers are not only consuming “milk tea”, but also “milk tea space” and “milk tea scene”. Therefore, the study of the distribution characteristics of this new type of consumer space and its influencing factors has important reference value for the layout and planning of the new type of urban retail consumer space. The research method of this paper can also be applied to the spatial distribution of other retail stores, but as a new type of urban consumer space, consumer landscape and consumer symbols, the study of milk tea stores is more typical and realistic. In the future, further research will be done on the transformation of urban consumption space.

## Supporting information

S1 Data Dataset used in the research https://doi.org/10.3886/E214781V1. (XLS)
